# Supplementary material for: Complex structural variation, phylogeny, and disease associations of the mucin pangenome
Source: medRxiv. 2026 Jul 4:2026.07.01.26356476. Preprint. [Version 1] doi: 10.64898/2026.07.01.26356476 (PMC13345508; doi:10.64898/2026.07.01.26356476)
Supplement: Supplementary file 1 [file NIHPP2026.07.01.26356476V1-supplement-1.pdf]

medRxiv preprint doi: https://doi.org/10.64898/2026.07.01.26356476; this version posted July 4, 2026. The copyright holder for this preprint (which was not certified by peer review) is the author/funder, who has granted medRxiv a license to display the preprint in perpetuity.
It is made available under a CC-BY 4.0 International license .

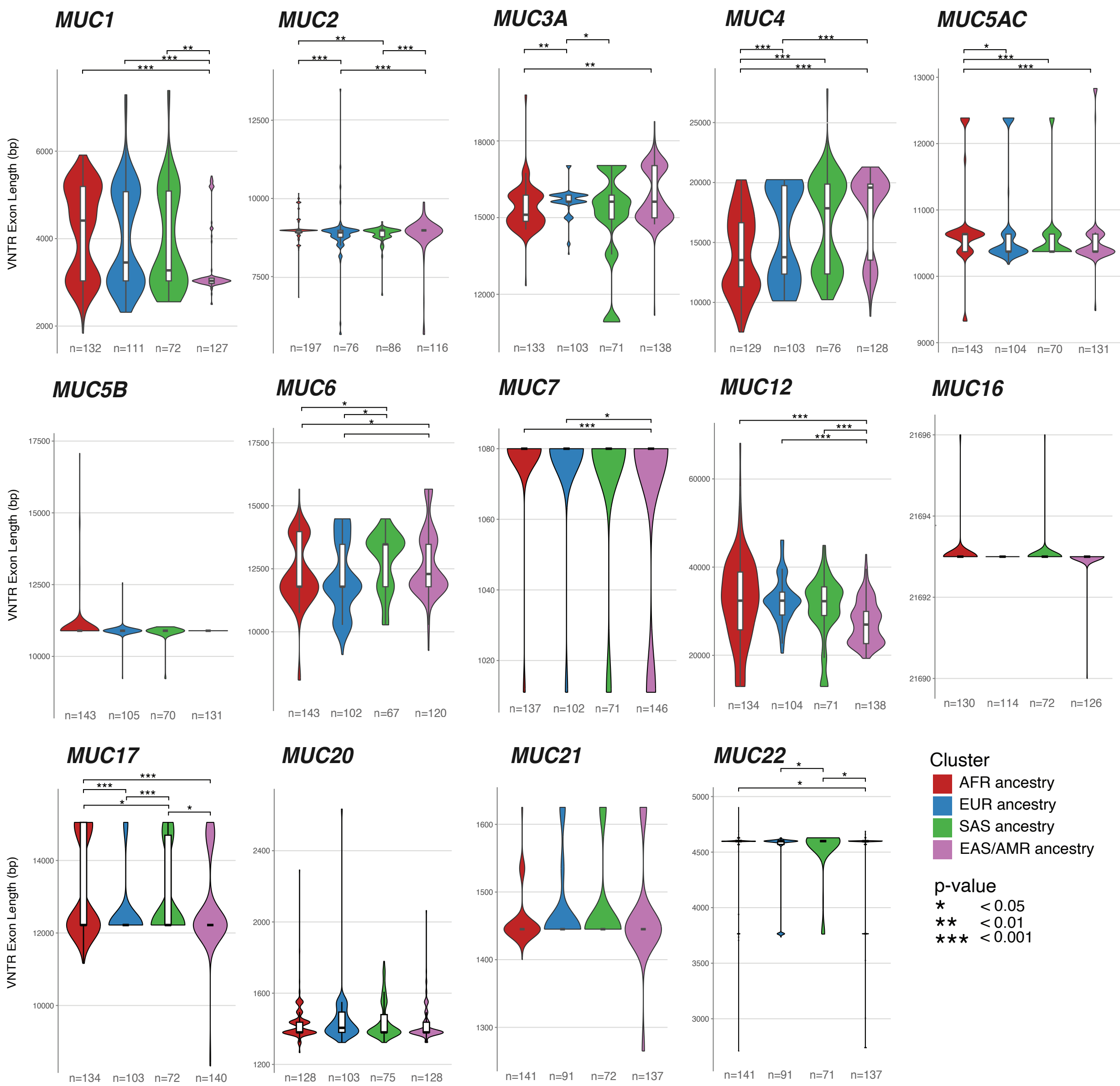

Supplementary Figure 1 | Mucin allele distributions with overlayed boxplots.
P-values correspond to pairwise Wilcoxon rank-sum tests with multiple testing correction (FDR). n corresponds to number of haplotypes (HPRC).

medRxiv preprint doi: https://doi.org/10.64898/2026.07.01.26356476; this version posted July 4, 2026. The copyright holder for this preprint (which was not certified by peer review) is the author/funder, who has granted medRxiv a license to display the preprint in perpetuity.
It is made available under a CC-BY 4.0 International license .

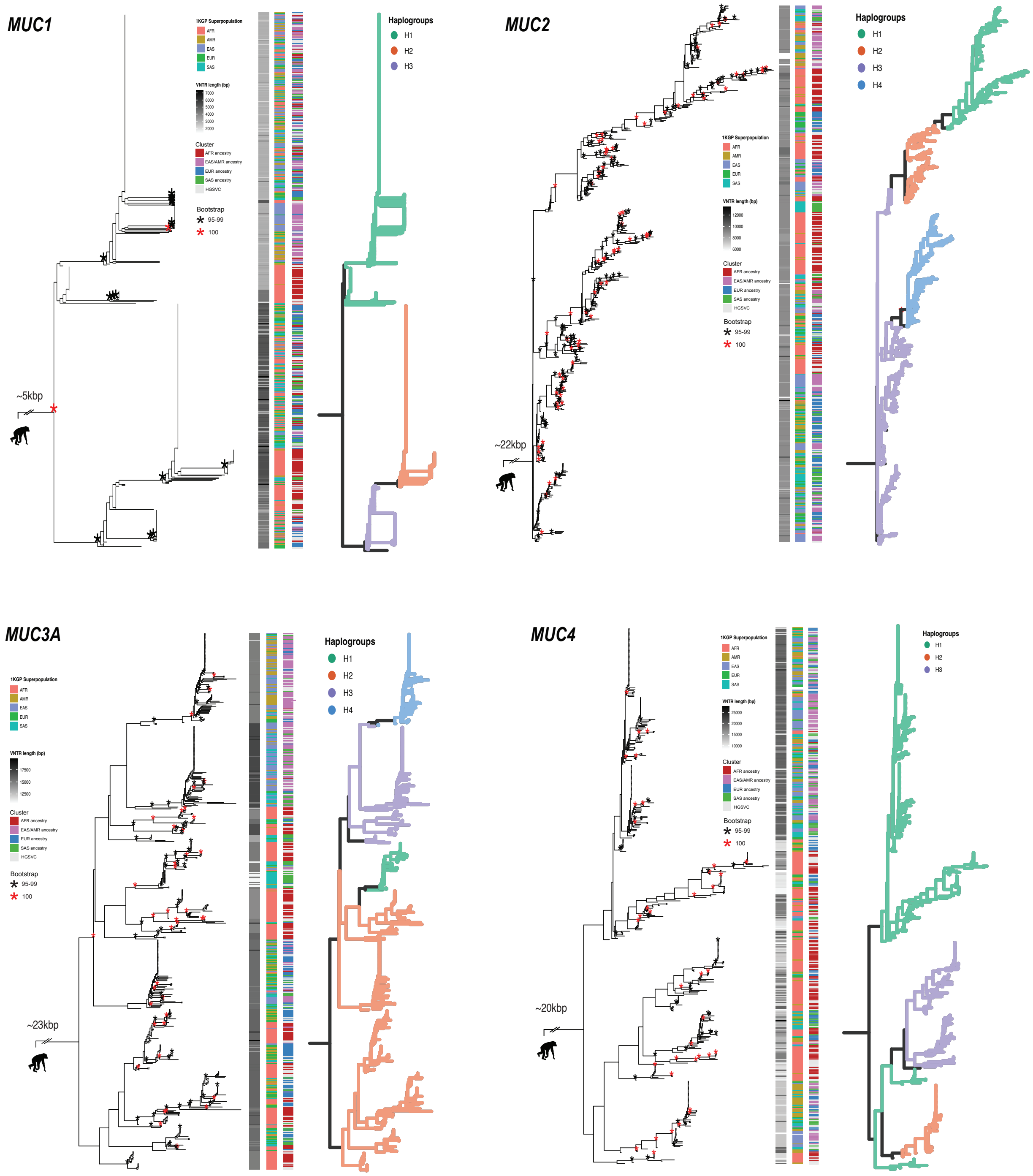

Supplementary Figure 2 | Complete phylogenetic trees and haplogroup overlay for *MUC1*, *MUC2*, *MUC3A*, and *MUC4*.
Two chimpanzee haplotypes were used as outgroup.

medRxiv preprint doi: https://doi.org/10.64898/2026.07.01.26356476; this version posted July 4, 2026. The copyright holder for this preprint (which was not certified by peer review) is the author/funder, who has granted medRxiv a license to display the preprint in perpetuity.
It is made available under a CC-BY 4.0 International license .

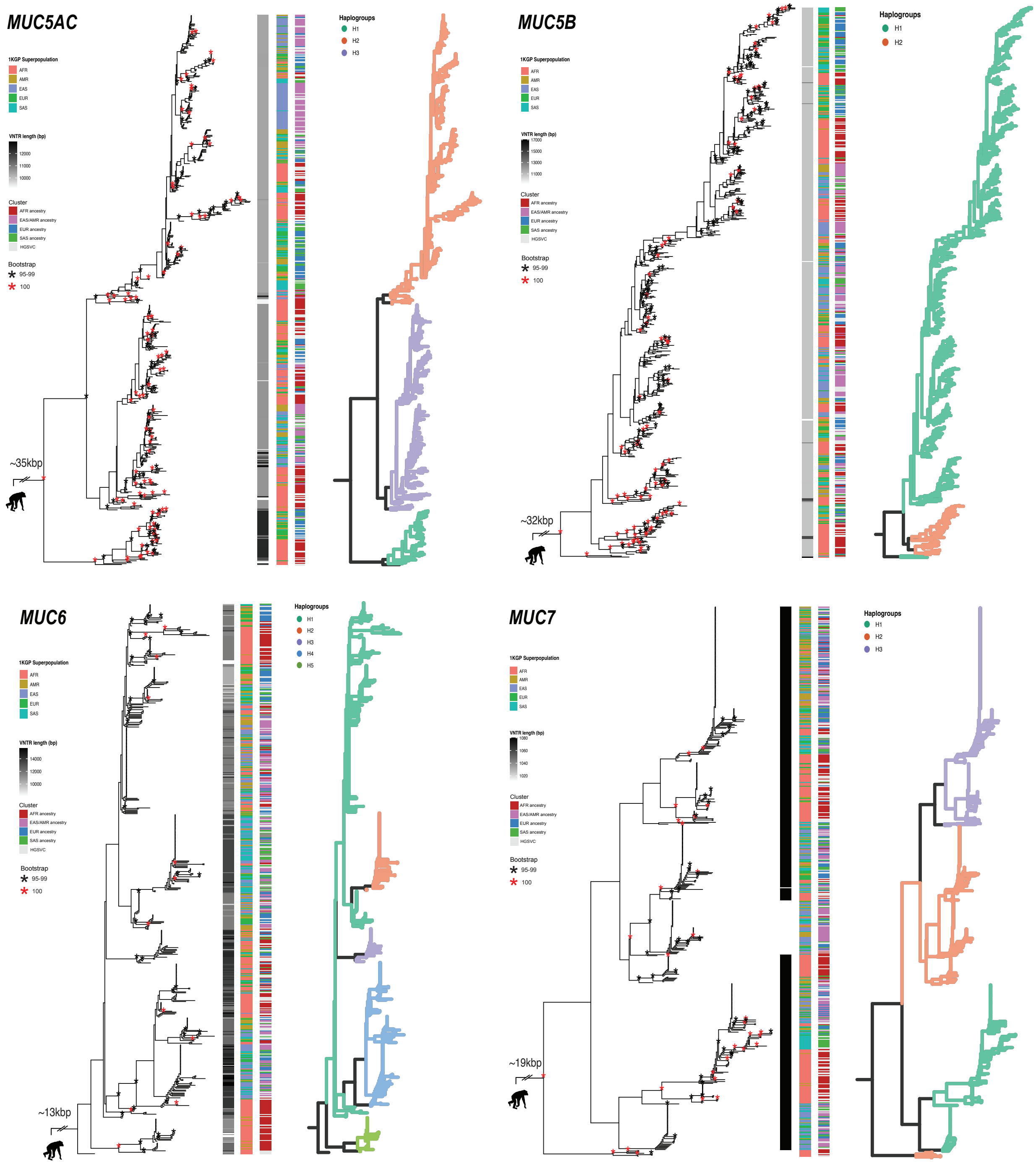

Supplementary Figure 3 | Complete phylogenetic trees and haplogroup overlay for *MUC5AC*, *MUC5B*, *MUC6*, and *MUC7*. Two chimpanzee haplotypes were used as outgroup.

medRxiv preprint doi: https://doi.org/10.64898/2026.07.01.26356476; this version posted July 4, 2026. The copyright holder for this preprint
(which was not certified by peer review) is the author/funder, who has granted medRxiv a license to display the preprint in perpetuity.
It is made available under a CC-BY 4.0 International license .

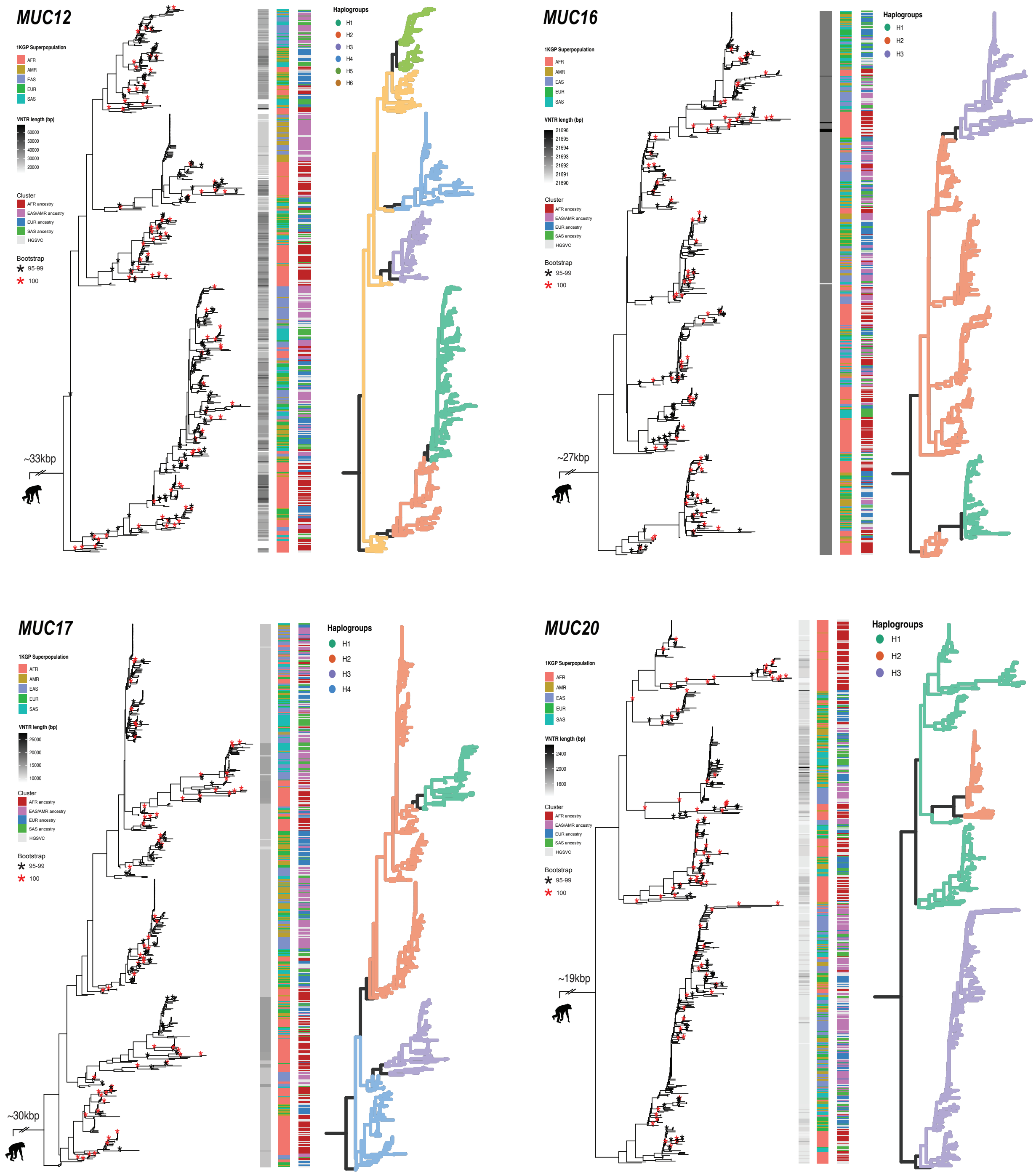

Supplementary Figure 4 | Complete phylogenetic trees and haplogroup overlay for *MUC12*, *MUC16*, *MUC17*, and *MUC20*. Two chimpanzee haplotypes were used as outgroup.

medRxiv preprint doi: https://doi.org/10.64898/2026.07.01.26356476; this version posted July 4, 2026. The copyright holder for this preprint (which was not certified by peer review) is the author/funder, who has granted medRxiv a license to display the preprint in perpetuity.
It is made available under a CC-BY 4.0 International license .

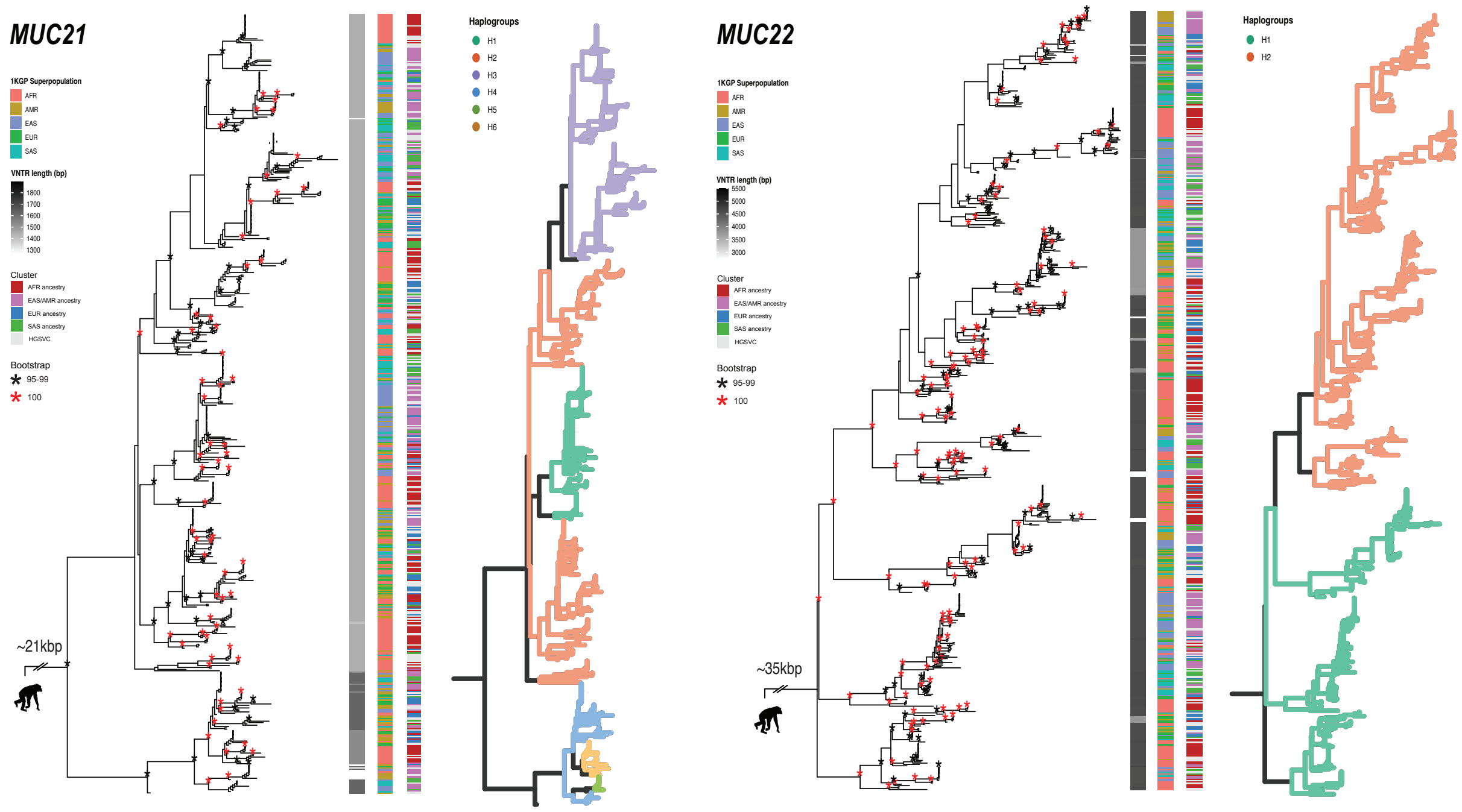

Supplementary Figure 5 | **Complete phylogenetic trees and haplogroup overlay for *MUC21* and *MUC22*.** Two chimpanzee haplotypes were used as outgroup.

medRxiv preprint doi: https://doi.org/10.64898/2026.07.01.26356476; this version posted July 4, 2026. The copyright holder for this preprint (which was not certified by peer review) is the author/funder, who has granted medRxiv a license to display the preprint in perpetuity.
It is made available under a CC-BY 4.0 International license .

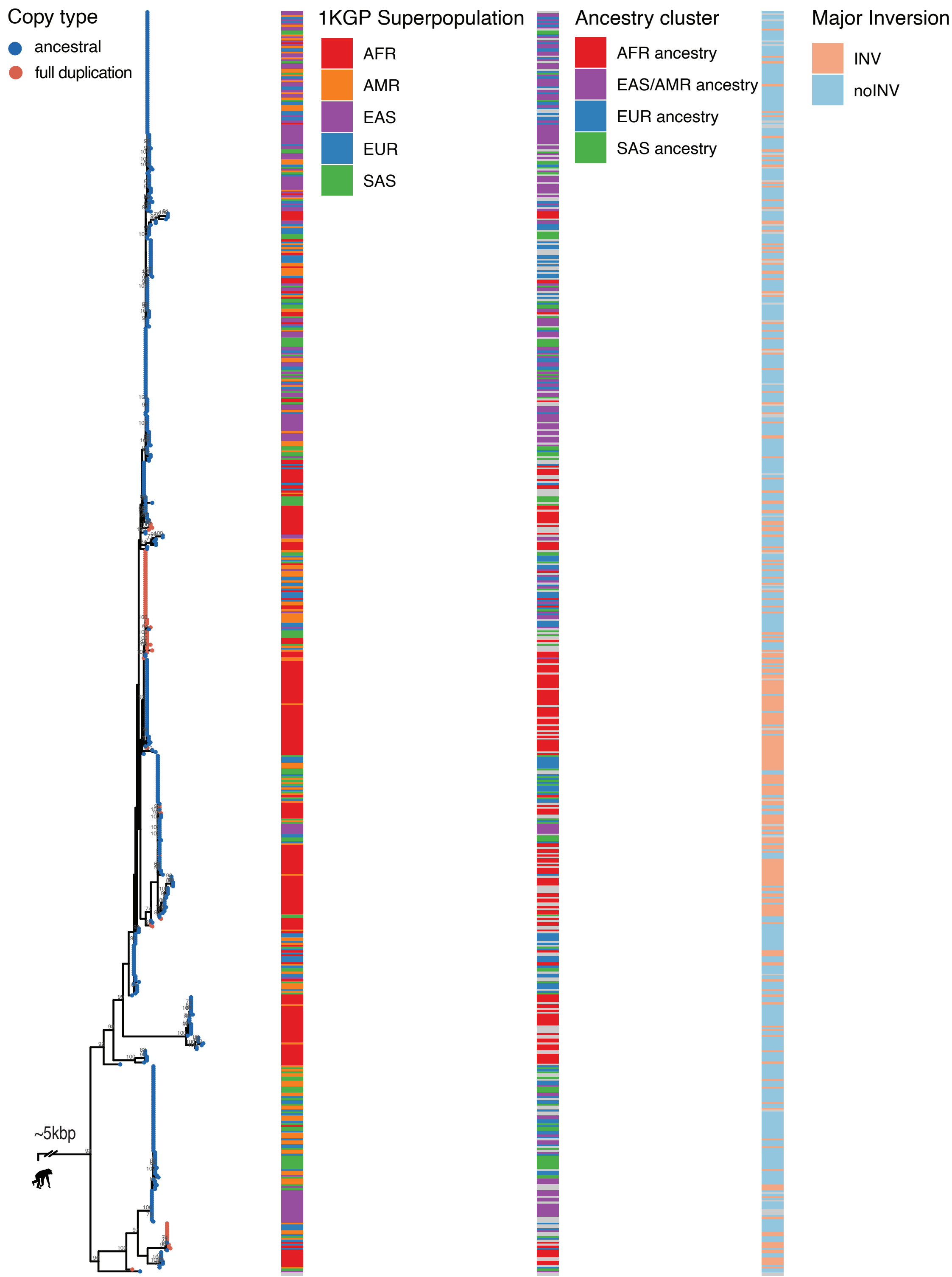

Supplementary Figure 6 | Phylogenetic tree of *MUC20* complete ancestral and full duplication copies in humans constructed without gene-converted sequence tracks.
Two chimpanzee haplotypes used as outgroup. Numbers at nodes of tree correspond to bootstrap support. Gene conversion tracks determined via GENECONV results and removed from alignment before tree construction. Major inversion signifies presence or absence of inverted region overlapping with *MUC4* and *MUC20*.

medRxiv preprint doi: https://doi.org/10.64898/2026.07.01.26356476; this version posted July 4, 2026. The copyright holder for this preprint (which was not certified by peer review) is the author/funder, who has granted medRxiv a license to display the preprint in perpetuity.
It is made available under a CC-BY 4.0 International license .

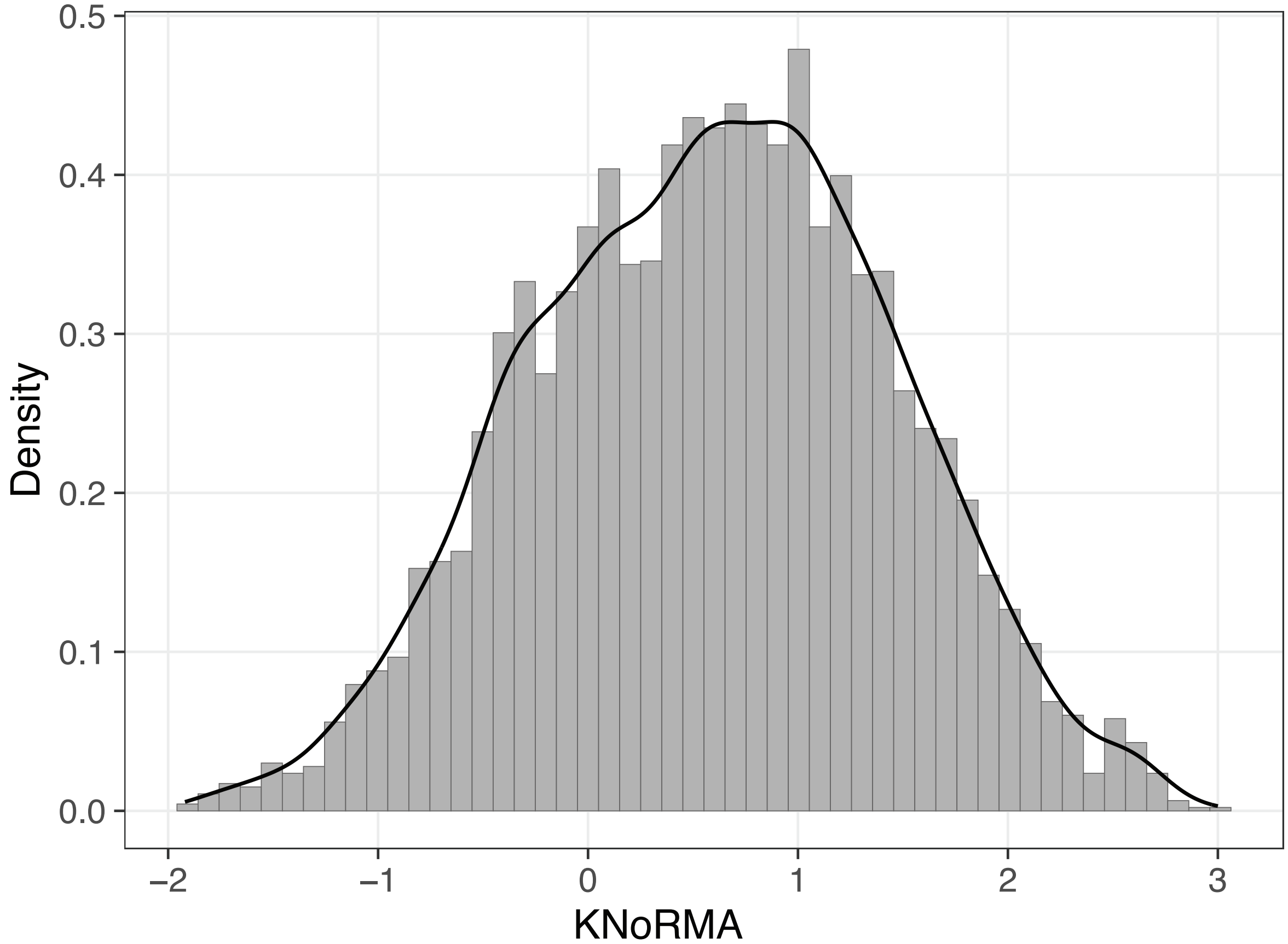

Supplementary Figure 7 | Distribution of KNoRMA scores across 4,637 paired short-read WGS data.

medRxiv preprint doi: https://doi.org/10.64898/2026.07.01.26356476; this version posted July 4, 2026. The copyright holder for this preprint (which was not certified by peer review) is the author/funder, who has granted medRxiv a license to display the preprint in perpetuity.
It is made available under a CC-BY 4.0 International license .

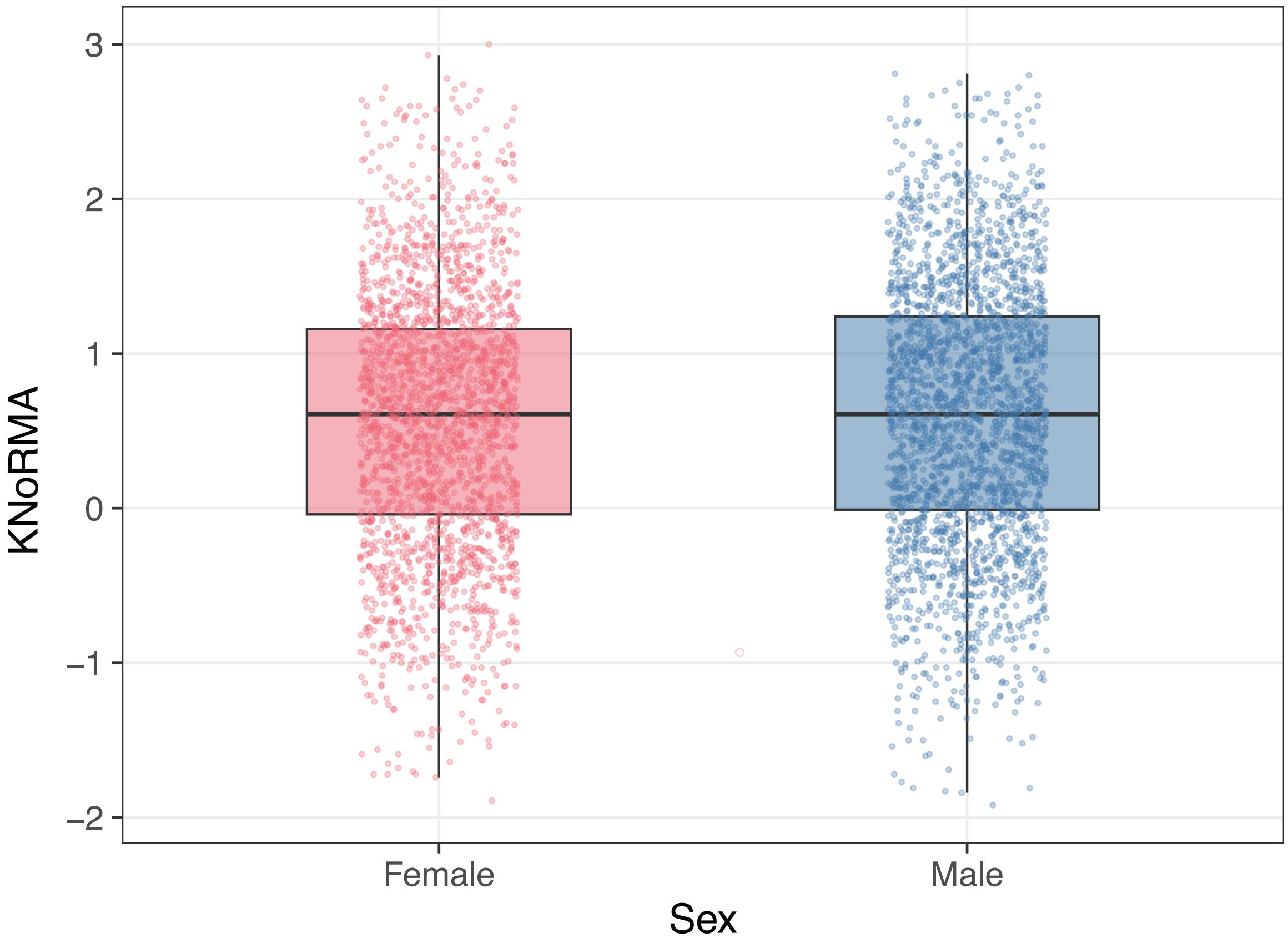

Supplementary Figure 8 | Sex distribution of KNoRMA scores across 4,637 paired short-read WGS data.

medRxiv preprint doi: https://doi.org/10.64898/2026.07.01.26356476; this version posted July 4, 2026. The copyright holder for this preprint (which was not certified by peer review) is the author/funder, who has granted medRxiv a license to display the preprint in perpetuity.
It is made available under a CC-BY 4.0 International license .

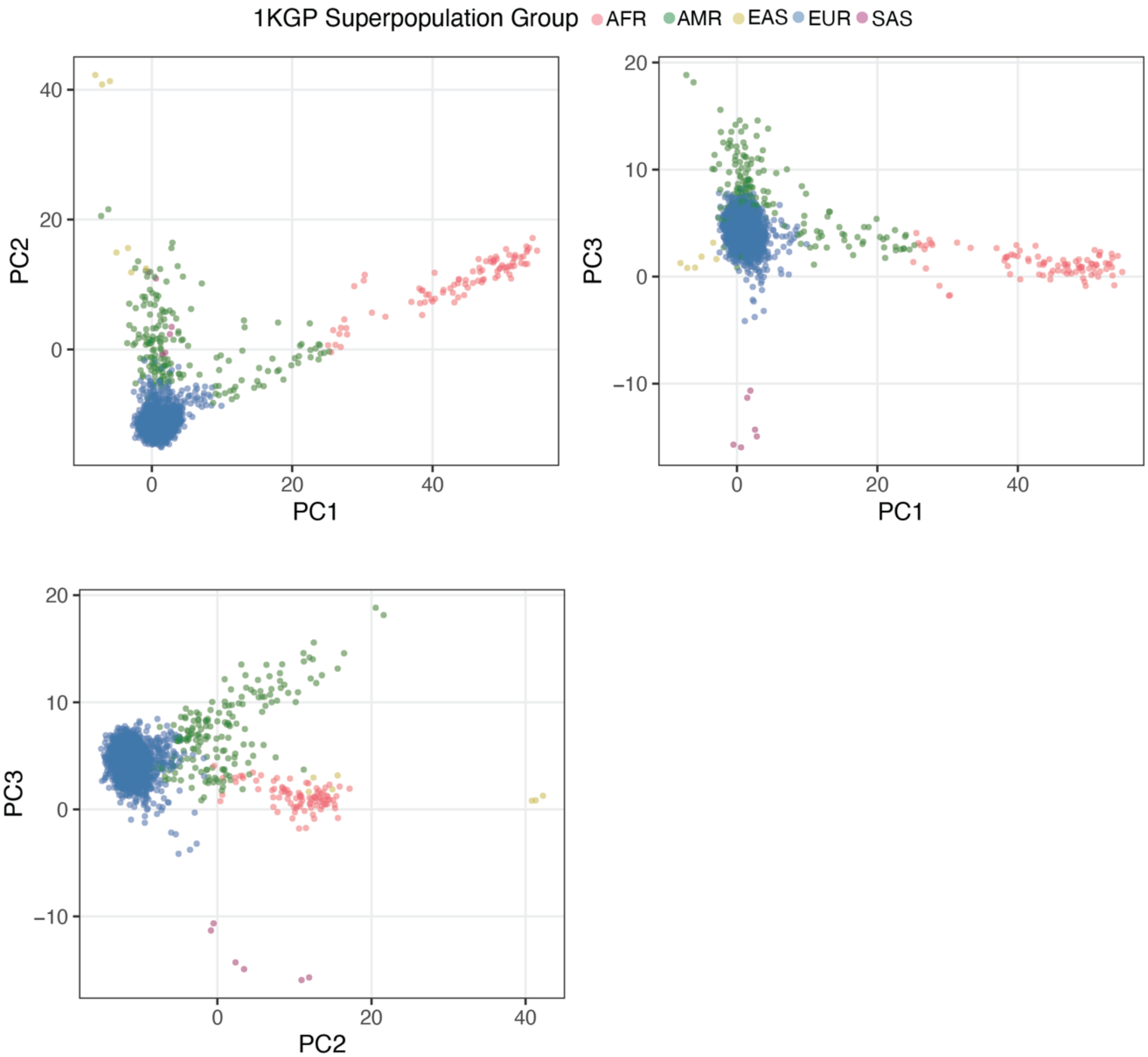

Supplementary Figure 9 | Principal components 1-3 from projection of 4,637 CF genomes onto 1KGP super-population labels.
